# The Vermont oxford neonatal encephalopathy registry: rationale, methods, and initial results

**DOI:** 10.1186/1471-2431-12-84

**Published:** 2012-06-22

**Authors:** Robert H Pfister, Peter Bingham, Erika M Edwards, Jeffrey D Horbar, Michael J Kenny, Terrie Inder, Karin B Nelson, Tonse Raju, Roger F Soll

**Affiliations:** 1 University of Vermont, Burlington, VT, USA; 2 Vermont Oxford Network, Burlington, VT, USA; 3 Washington University, St Louis, MO, USA; 4 National Institute of Neurological Disorders and Stroke, Bethesda, MD, USA; 5 Children's Hospital National Medical Center, Washington, DC, USA; 6 National Institute of Child Health and Human Development, Bethesda, MD, USA; 7 Vermont Oxford Network Neonatal Encephalopathy Registry Steering Committee Member, Burlington, USA

**Keywords:** Hypoxic ischemic encephalopathy, Neonatal encephalopathy, HIE, Therapeutic hypothermia, Asphyxia, Cooling, Neuroprotection, Neonatal encephalopathy, Registry

## Abstract

**Background:**

In 2006, the Vermont Oxford Network (VON) established the Neonatal Encephalopathy Registry (NER) to characterize infants born with neonatal encephalopathy, describe evaluations and medical treatments, monitor hypothermic therapy (HT) dissemination, define clinical research questions, and identify opportunities for improved care.

**Methods:**

Eligible infants were ≥ 36 weeks with seizures, altered consciousness (stupor, coma) during the first 72 hours of life, a 5 minute Apgar score of ≤ 3, or receiving HT. Infants with central nervous system birth defects were excluded.

**Results:**

From 2006–2010, 95 centers registered 4232 infants. Of those, 59% suffered a seizure, 50% had a 5 minute Apgar score of ≤ 3, 38% received HT, and 18% had stupor/coma documented on neurologic exam. Some infants experienced more than one eligibility criterion. Only 53% had a cord gas obtained and only 63% had a blood gas obtained within 24 hours of birth, important components for determining HT eligibility. Sixty-four percent received ventilator support, 65% received anticonvulsants, 66% had a head MRI, 23% had a cranial CT, 67% had a full channel encephalogram (EEG) and 33% amplitude integrated EEG. Of all infants, 87% survived.

**Conclusions:**

The VON NER describes the heterogeneous population of infants with NE, the subset that received HT, their patterns of care, and outcomes. The optimal routine care of infants with neonatal encephalopathy is unknown. The registry method is well suited to identify opportunities for improvement in the care of infants affected by NE and study interventions such as HT as they are implemented in clinical practice.

## Background

Neonatal encephalopathy (NE) in the term or late preterm infant is "a clinically defined syndrome of disturbed neurologic function in the earliest days of life manifested by difficulty with initiating and maintaining respiration, depression of tone and reflexes, subnormal level of consciousness, and often by seizures" [1]. NE occurs in an estimated 2–5 per 1000 live term births of which up to one quarter experience moderate or severe cerebral injury [2-4]. Between 10-40% do not survive and as many as 30% exhibit significant long-term neurodevelopmental disability [5].

Randomized controlled trials (RCTs) demonstrated that hypothermic therapy (HT) may improve neurologic and developmental outcomes and reduce death and disability in term infants with NE [6-9]. As a result, many practitioners have lost equipoise [10,11]. The National Institute of Child Health and Human Development and the American Academy of Pediatrics Committee on Fetus and Newborn caution that clinicians should follow published trial protocols, ensure systematic follow-up of survivors, and submit patient data to registries when using HT outside of a trial [12,13]. Registries, by documenting the natural history of enrolled patients as they present for care, monitor clinical patterns and patient outcomes in rare disorders such as NE and track the “real world” dissemination of a novel therapy like HT [14]. 

The Vermont Oxford Network (VON) is a non-profit voluntary collaboration of health care professionals dedicated to improving the quality and safety of medical care for newborn infants and their families at over 850 neonatal intensive care units (NICU) around the world. The VON Neonatal Encephalopathy Registry (NER) was established in 2006.

The primary objective is to characterize infants born with NE, including perinatal and antenatal risk factors, how these infants are identified, the evaluations and treatments they receive, and their outcomes. Secondary objectives include monitoring the dissemination and uptake of the novel therapies such as HT and description of variation of care applied to NE infants. These data will help define clinical research questions and identify opportunities for improved care of NE. This manuscript describes the methods and basic demographic results of the VON NER.

## Methods

Hospitals could enroll patients in the NER through participation in one of two databases maintained by VON. The very low birth weight (VLBW) database includes any infant born alive at a participating hospital with a birth weight 401–1500 grams or a gestational age of 22–29 weeks regardless of where the infant receives care, as well as any outborn infant meeting these criteria admitted to any location in the hospital within 28 days of birth without first having gone home. The Expanded database includes any infant regardless of birth weight or gestational age admitted to the hospital’s NICU by day 28.

In 2006 and 2007, only VON Expanded database centers could participate in the NER. Beginning in 2008, all VON database participating centers were eligible. Participation in the NER requires no additional fee. VON uses these data for research and reporting, but maintains the confidentiality of individual hospital data. Participating hospitals receive reports comparing their local data with the Registry as a whole. A participating NER center submitted data on one or more eligible infants.

### Infant eligibility

**Figure 1:**
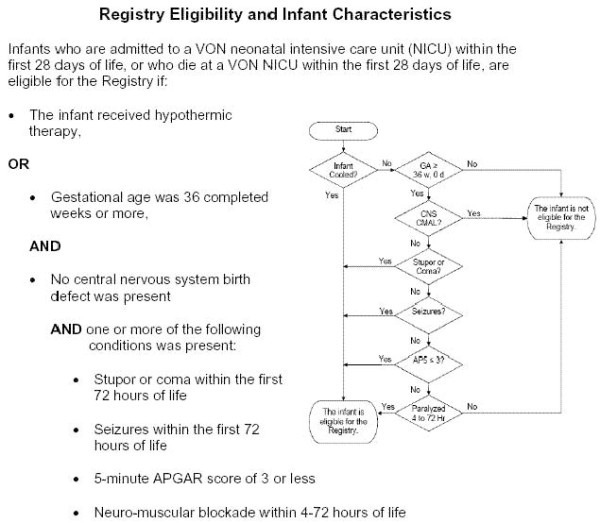
Registry Eligibility and Infant Characteristics.

Any infant born at 36 weeks gestation or more displaying evidence of NE within 3 days of birth is eligible. NE is defined as presence of seizures and/or altered consciousness (stupor, coma). In order to cast a wide net that captures all infants potentially affected by NE independent of the adequacy of their neurologic exam, infants with a 5 minute Apgar score of ≤ 3 are included. Accordingly, infants that received neuromuscular blockade are also eligible since their level of conscious could not be assessed. Regardless of neurologic status, any infant that received HT is eligible. Infants born with central nervous system (CNS) birth defects are excluded (Figure 1).

### Data items

The VON NER Steering Committee chose data items to characterize the population of all infants with NE, identify potential antecedents, evaluate variations in current practice, and monitor the dissemination of HT and adherence to the RCT efficacy standards. Data items include: patient identifiers, patient selection criteria, infant characteristics, treatments and tests, and outcomes at time of disposition. Where possible, data forms follow standards and terminology derived from existing studies to contribute to evolving medical knowledge. Participating centers receive explicit data definitions for each variable to ensure internal validity and uniform data acquisition. A complete catalogue of data items and definitions are in the manual of operations published on the VON website: http://www.vtoxford.org/tools/downloads.aspx.

Centers collect and submit data using freely provided VON eNICQ software, which provides easy to use on-screen data definitions, immediate feedback on issues such as missing or out-of-range values, and error checking for logical inconsistencies. VON staff members perform additional data assessment and contact hospitals about missing data items, unresolved records, out-of-range values, and appropriate modifications as indicated. Only de-identified data are submitted to VON.

The Registry does not dictate patient care, propose any interventions, or endorse any protocols for treatment. Each infant receives care according to the standards of that institution. There is no expected increased risk for participation of individual patients and only de-identified data are submitted. The University of Vermont and State Agricultural College Committee on Human Research in the Medical Sciences (CHRMS) Institutional Review Board (IRB) at the University of Vermont granted ethical approval for the methods of the NER (reference number CHRMS 06–100). Additionally, participating hospitals gained local IRB approval for the participation in the Registry. VON requires documentation of each participating center’s local IRB approval before participation in the Registry. Submitted data becomes the property of VON. The Network may use these data for research and reporting, but maintains the confidentiality of individual hospital data.

Outcomes of interest in the NER include death prior to hospital discharge, survivor disposition status, neurologic course, presence of seizures, common neonatal co-morbidities, and adverse events associated with HT including the following: arrhythmia, thrombosis, severe hypotension, seizure during re-warming, scalp edema, skin breakdown, sclerema neonatorum, thrombocytopenia, and infection. These outcomes will be addressed in future NER studies.

### Data analysis

We summarized demographic and clinical characteristics with percentages for categorical variables, mean (and standard deviation) for normally distributed variables, and median (and interquartile range) for other continuous variables. Hospital characteristics come from the VON Annual Survey.

## Results

### Hospital participation

From 2006 to 2010, 95 centers registered infants in the NER (Table 1). Participating hospitals averaged 686 (Quartile 1 (Q1): 473, Quartile 3 (Q3): 830) annual NICU admissions. A complete list of participating hospitals is presented in Table 2. We averaged each center’s annual volume across all of the years in which the center submitted NER records. The mean number of infants that met eligibility requirements per center was 44.5 (Q1: 15.0, Q3: 57.0).

**Table 1:** Growth of Vermont Oxford Network National Encephalopathy Registry: Participating centers and infants per year, 2006-2010

**Year of birth**	**Number of centers**	**Number of infants**
2006	41	433
2007	37	547
2008	62	813
2009	87	1229
2010	85	1210
2006-2010	95	4232

**Table 2:** Hospitals registering infants in the Vermont Oxford Network Neonatal Encephalopathy Registry, 2006–2010

Name	*City*	*State*	*Country*
Cork University Maternity Hospital	Cork		Ireland
National Maternity Hospital	Dublin		Ireland
Rotunda Hospital	Dublin		Ireland
Hospital de S. Joao	Porto		Portugal
Hospital Sant Joan de Deu	Barcelona		Spain
Latifa Hospital	Dubai		United Arab Emirates
Southmead Hospital	Bristol		United Kingdom
Arkansas Children's Hospital	Little Rock	Arkansas	United States
UC Irvine Medical Center	Orange	California	United States
Sharp Mary Birch Hospital for Women	San Diego	California	United States
Santa Clara Valley Medical Center	San Jose	California	United States
The Children's Hospital	Aurora	Colorado	United States
Exempla St. Joseph Hospital	Denver	Colorado	United States
Poudre Valley Health System	Fort Collins	Colorado	United States
Yale New Haven Children's Hospital	New Haven	Connecticut	United States
Christiana Care Health Services	Newark	Delaware	United States
Children's Hospital of SW Florida at Lee Memorial	Fort Myers	Florida	United States
Baptist Children's Hospital	Miami	Florida	United States
Miami Children's Hospital	Miami	Florida	United States
St. Joseph's Children's Hospital of Tampa	Tampa	Florida	United States
Tampa General Hospital	Tampa	Florida	United States
Medical Center at Columbus Regional, The	Columbus	Georgia	United States
St. Luke's Regional Medical Center	Boise	Idaho	United States
Evanston Hospital	Evanston	Illinois	United States
Edward Hospital and Health Services	Naperville	Illinois	United States
Advocate Lutheran General Hospital	Park Ridge	Illinois	United States
Rockford Memorial Hospital	Rockford	Illinois	United States
St. John's Hospital	Springfield	Illinois	United States
Carle Foundation Hospital	Urbana	Illinois	United States
Central DuPage Hospital	Winfield	Illinois	United States
St. Luke's Hospital	Cedar Rapids	Iowa	United States
Blank Children's Hospital	Des Moines	Iowa	United States
Overland Park Regional Medical Staff	Overland Park	Kansas	United States
Wesley Medical Center	Wichita	Kansas	United States
Kosair Children's Hospital	Louisville	Kentucky	United States
Woman's Hospital	Baton Rouge	Louisiana	United States
Eastern Maine Medical Center	Bangor	Maine	United States
Barbara Bush Children's at Maine Medical	Portland	Maine	United States
University of Maryland Division of Neonatology	Baltimore	Maryland	United States
Frederick Memorial Hospital	Frederick	Maryland	United States
Massachusetts General Hospital for Children	Boston	Massachusetts	United States
UMass Memorial Healthcare	Worcester	Massachusetts	United States
U. of MI, CS Mott Children's, Brandon NICU	Ann Arbor	Michigan	United States
Henry Ford Hospital	Detroit	Michigan	United States
DeVos Children's, Spectrum Health	Grand Rapids	Michigan	United States
Sparrow Hospital	Lansing	Michigan	United States
University of MN Children's Hospital, Fairview	Minneapolis	Minnesota	United States
North Memorial Medical Center	Robbinsdale	Minnesota	United States
St. Cloud Hospital	Saint Cloud	Minnesota	United States
St. Francis Medical Center, Cape Girardeau	Cape Girardeau	Missouri	United States
Cardinal Glennon Children's Hospital	St. Louis	Missouri	United States
St. Louis Children's Hospital	St. Louis	Missouri	United States
St. Elizabeth Regional Medical Center	Lincoln	Nebraska	United States
Alegent Health Bergen Mercy Medical Center	Omaha	Nebraska	United States
Nebraska Medical Center	Omaha	Nebraska	United States
Albany Medical Center	Albany	New York	United States
Weiler Hospital Montefiore	Bronx	New York	United States
Winthrop University Hospital	Mineola	New York	United States
Columbia University Medical Center	New York	New York	United States
Golisano Children's Hospital at Strong	Rochester	New York	United States
Mission Children's Hospital	Asheville	North Carolina	United States
Duke University	Durham	North Carolina	United States
Cape Fear Valley Medical Center	Fayetteville	North Carolina	United States
Women's Hospital of Greensboro	Greensboro	North Carolina	United States
Pitt County Memorial Hospital	Greenville	North Carolina	United States
WAKEMED Faculty Physicians, Wake Medical Center	Raleigh	North Carolina	United States
Brenner Children's Hospital at WFUBMC	Winston-Salem	North Carolina	United States
Akron Children's Hospital	Akron	Ohio	United States
Children's Hospital Medical Center Cincinnati	Cincinnati	Ohio	United States
Henry Zarrow Neonatal Intensive Care Unit	Tulsa	Oklahoma	United States
Rogue Valley Medical Center	Medford	Oregon	United States
Providence St. Vincent Medical Center	Portland	Oregon	United States
Randall Children's Hospital at Legacy Emanuel	Portland	Oregon	United States
Salem Hospital	Salem	Oregon	United States
Sacred Heart Medical Center	Springfield	Oregon	United States
St. Luke's University Hospital	Bethlehem	Pennsylvania	United States
Geisinger Medical Center	Danville	Pennsylvania	United States
Penn State Children's Hospital	Hershey	Pennsylvania	United States
Thomas Jefferson University Hospital	Philadelphia	Pennsylvania	United States
Magee Women's Hospital	Pittsburgh	Pennsylvania	United States
Palmetto Health Richland	Columbia	South Carolina	United States
Children's Hospital of Greenville	Greenville	South Carolina	United States
University of Tennessee Medical Center	Knoxville	Tennessee	United States
Baptist Memorial Hospital for Women	Memphis	Tennessee	United States
Monroe Carell Jr. Children's Hospital Vanderbilt	Nashville	Tennessee	United States
Cook Children's Medical Center	Fort Worth	Texas	United States
Christus Santa Rosa Healthcare	San Antonio	Texas	United States
Methodist Children's Hospital	San Antonio	Texas	United States
Vermont Children's at Fletcher Allen Health Care	Burlington	Vermont	United States
Carilion Clinic Children's Hospital	Roanoke	Virginia	United States
Swedish Medical Center	Seattle	Washington	United States
West Virginia University School of Medicine	Morgantown	West Virginia	United States
Gundersen Lutheran Medical Center	LaCrosse	Wisconsin	United States
St. Mary's Hospital Medical Center	Madison	Wisconsin	United States
Wheaton Franciscan Healthcare at St. Joseph	Milwaukee	Wisconsin	United States

Almost all (97%) NER centers were non-profit (Table 3). Minority-serving hospitals, those that treat >35% black infants, [15] constituted 18% of the participating hospitals. Over three-fourths of the participating centers had pediatric residents or neonatology fellows working within their NICUs. Almost all centers had MRI scanning capability. 

**Table 3:** Hospital Characteristics in Vermont Oxford Network Neonatal Encephalopathy Registry

Characteristic*	**Number of hospitals**	**%**
Non-profit	92	96.8
Minority Serving Hospital	17	17.9
Teaching Hospital	72	75.8
Children’s Hospital	15	15.8
MRI Scanning Capability	92	96.8
AAP Level IIIA	9	9.5
AAP Level IIIB	49	51.6
AAP Level IIIC and IIID	33	34.7
AAP Level Unknown	4	4.2

*Based on the last year in which the center submitted NER data.

VON classifies participating NICUs using a method based on the AAP Levels of Neonatal Care classification set forth by the Committee on Fetus and Newborn [16]. The VON annual survey does not differentiate between Level IIIC (those that provide major surgical services excluding serious congenital heart anomalies that require cardiopulmonary bypass or extracorporeal membrane oxygenation (ECMO)) and Level IIID hospitals (those that do provide major surgery including surgical repair of serious congenital heart anomalies or ECMO). All NER hospitals classified themselves in the annual survey as subspecialty intensive care (level III) hospitals. The majority (52%) were level IIIB hospitals, which have no restrictions on the duration of mechanical ventilation but do not provide major surgery.

### Infant eligibility

Of the 4232 eligible infants, 59% suffered a clinically apparent seizure within the first 72 hours of life, 50% had a 5 minute Apgar score of 3 or less, 38% had HT, 18% had stupor/coma, and 2% had neuromuscular blockade. HT as the sole eligibility criteria accounted for 8% of the entire sample. Many infants (39%) experienced more than one eligibility criterion. Among infants with multiple eligibility criteria, 30.7% received hypothermia, 28.1% had an Apgar score of 3 or less, 26.9% had a clinically apparent seizure, 17.2% had stupor or coma, and only 1.2% had neuromuscular blockade.

### Infant characteristics

Registered infants had a median birth weight of 3298 grams (Q1: 2905, Q3: 3685) and a median gestational age of 39 weeks (Q1: 38, Q3: 40). Over one-third of infants were not admitted to the NICU until 6 hours after birth (Table 4). Of those not admitted until after 6 hours, 81% were outborn. Sixteen percent were small for gestational age. Over 60% of infants required transport. Over half (56%) were delivered by cesarean section (C/S), the majority of which had a trial of labor before the C/S. Fourteen percent of infants had a traumatic birth injury. A cord gas was obtained at the time of delivery in 53% of enrolled infants. Of those obtained, the mean pH was 7.0 (Q1: 6.9, Q3: 7.2) and the mean cord gas base excess was −12.2 (Q1: -17.6, Q3: -6.0).

**Table 4:** Characteristics of Infants in Vermont Oxford Network National Encephalopathy Registry, 2006-2010

	**Eligible**	**N**	**%**
Admission Time > 6hrs	4165	1395	33.5
Small for Gestational Age	4231	676	16.0
Inborn	4232	1682	39.7
Maternal Race	4194		
Asian		95	2.3
Black		777	18.5
Hispanic		574	13.7
White		2675	63.8
Other		73	1.7
Number of Births	4232		
Singleton		4167	98.5
Twins		65	1.5
Delivery Method	4228		
Spontaneous vaginal		1414	33.4
Vaginal delivery using vacuum/forceps		450	10.6
Cesarean section before labor		840	19.9
Cesarean section after labor		1524	36.1
Traumatic Birth Injury	4206	590	14.0
Meconium Aspiration Syndrome	4227	515	12.2
Cord Gas Obtained*	3699	1946	52.6

*The NER specified arterial cord blood gas from 2006–2008 but in 2009–2010 included umbilical cord blood from any source.

### Evaluations and treatments

Of NER infants, 64% received ventilator support, 38% received HT, and 65% received anticonvulsants for any indication (Table 5). Thirteen percent received inhaled nitric oxide and 3% received ECMO. Approximately 9% of the infants had surgery during their hospitalization, mainly abdominal. Sixty-six percent of infants underwent a head MRI and 49% received a cranial ultrasound. Sixty-seven percent had a full channel encephalogram (EEG) while 33% underwent amplitude integrated EEG monitoring. Overall, 36% of infants did not have a blood gas obtained from any site (arterial, venous, or capillary). Of those infants with a value, the worst gas results yielded a mean pH of 7.1 (Q1: 7.0, Q3: 7.3) and a mean base excess of −13.0 (Q1: -20.0, Q3: -6.0).

**Table 5:** Evaluations and treatments received by infants in the Vermont Oxford Network National Encephalopathy Registry, 2006-2010

	**Elig**	**N**	**%**
Therapeutic hypothermia	4232	1626	38.4
Anticonvulsant during hospital course	4177	2715	65.0
Blood gas obtained within 24 hours^1^	1723	1083	62.9
Blood gas obtained within first hour^2^	2281	1462	64.1
Cranial ultrasound	4172	2045	49.0
Cranial CT	4168	951	22.8
MRI	4170	2742	65.8
Full channel EEG	4171	2777	66.6
Amplitude integrated EEG (aEEG)	4168	1376	33.0
High flow nasal cannula (HFNC)	4167	1371	32.9
Nasal CPAP	4168	748	18.0
Ventilator	4168	2723	65.3
High frequency oscillatory ventilation (HFOV)	4168	486	11.7
Extracorporeal membrane oxygenation (ECMO)	4167	118	2.8
Inhaled nitrous oxide (iNO)	4167	556	13.3
Any Surgery	4168	379	9.0
Cardiac	4168	22	0.5
PDA	4168	9	0.2
Abdominal	4168	301	7.2
CNS	4168	25	0.6

^1^Question for 2006–2008.

^2^Question for 2009–2010.

### Outcomes

Of all infants, 87% survived (Table 6). Among the survivors, at discharge 38% were on anticonvulsants, 86% received all feeds by mouth, 6% had home monitoring, and 1% had ventilator support. The typical length of stay among surviving infants discharged to home was 11 days (Q1: 7, Q3: 19). Of infants that died during their initial hospitalization, the median day of death was day 4 (Q1: 2, Q3: 9).

**Table 6:** Outcomes at initial disposition of all infants in the Vermont Oxford Network National Encephalopathy Registry, 2006-2010

	**Elig**	**N**	**%**
Survival status			
Died	4232	551	13.0
Alive	4232	3676	86.9
Unknown	4232	5	0.1
**Among Survivors**			
Anticonvulsants at discharge	3670	1393	38.0
Feeds at discharge			
Enteral, all by mouth	3670	3141	85.6
Enteral, none by mouth	3670	259	7.1
Some by mouth	3670	201	5.5
No enteral feeding	3670	69	1.9
Hearing screen passed	3212	2942	91.6
Discharged home			
On monitor	3439	220	6.4
On oxygen	3441	132	3.8
On ventilator	3676	50	1.4

## Discussion

A patient registry is an organized system that uses observational study methods to collect uniform data and evaluate specified outcomes for a population defined by a particular disease, condition, or exposure, and that serves a predetermined scientific, clinical, or policy purpose(s) [17]. Registries can support clinical conditions, health care services, or products, and can address questions ranging from treatment effectiveness and safety to the quality of care delivered.

The VON NER captures data and characterizes infants with NE and a subset treated with HT. To increase external validity, inclusion criteria for the VON NER are intentionally few and simple: the presence of seizures and/or altered consciousness (stupor, coma) during the first 72 hours of life. Additional inclusion parameters capture all potentially encephalopathic infants treated with hypothermia independent of their neurologic status and infants whose neurologic status might be difficult to assess (e.g., paralyzed, mechanically ventilated, or sedated infants).

Historically, the presence of NE has been considered *sine qua non* of hypoxic-ischemic injury or birth asphyxia. However, the etiology of NE is not limited to hypoxic-ischemic injury and displays considerable diversity [18]. Only a small proportion of infants in the NER had documented exposure to acute intrapartum asphyxia (“sentinel events”). These findings reflect previous research suggesting that a minority (25-35%) of cases of NE attributed to birth asphyxia have a clear contributing sentinel event in the intrapartum period [13,19]. The VON NER is being used describe the frequency with which recognized antecedents of NE occurred in a large sample of encephalopathic term newborns. These findings will have implications for future studies of the etiology of NE.

Timely recognition of NE infants affected by HIE is crucial to the success of HT. Very few infants in the Registry were identified as having altered consciousness on neurologic exam. In fact, the most common route for entry was following a seizure. Among the subset of NE infants caused by HIE, by the time an infant suffers seizures it may be too late to achieve the full benefit of HT [20,21]. Given that birth asphyxia is often presumed the etiology of NE, it is striking to note that umbilical cord blood gas examinations were obtained in just over half of the infants during their perinatal courses. Similarly, less than 40% of the infants had blood gas sampling performed following birth. Since neurologic exams and umbilical and cord blood gas examinations are commonly used tests to determine whether or not HT is appropriate, these data suggest there is room for improvement in the recognition and evaluation of NE.

Nearly four in ten (38%) NER infants were eligible due to exposure to HT. However the majority were also encephalopathic and in only a small percentage (8%) was HT the sole criteria for eligibility. One third of infants receiving HT were admitted after 6 hours of life when any neuroprotective benefit from HT may be diminished. Over 60% of NE infants required transport, which may be a significant contributing factor in the observed delay in admission. Identification of gaps between the conditions for implementation of HT in clinical trials and in what is observed in clinical practice and identification of areas of improvement are focused areas of ongoing NER research.

The optimal routine evaluation and treatments of infants with NE is unknown. The majority of infants were treated with anticonvulsants but significant variation was noted in the approach to electroencephalographic monitoring. Only 66% had optimal imaging (head MRI) and 23% underwent a suboptimal exam (head CT) according to accepted quality standards [22]. Ongoing work of the NER will identify and document variation in the evaluations and medical treatments these infants receive, providing valuable information for future RCTs.

Variation in patient selection and adherence to established protocols contributes to differences in survival, adverse events, and long-term outcomes for treated infants [23,24]. Among infants in the NER, 13% died, a proportion similar to the mortality rate of NE observed in a population based report by Badawi et al. (9%) despite different inclusion criteria [3]. Of infants that survived to discharge, a significant proportion required ventilation, monitoring, and other medical care at home. These findings are consistent with previous observations of the medical burden and mortality associated with NE infants and underscores the need for improvement of the quality of care. The NER provides benchmarking data that member centers use while participating in VON multicenter quality improvement collaborations.

A large proportion of cerebral palsy, cognitive disability, and epilepsy arise in infants born at term or late preterm [25]. In contrast to preterm infant births, the births of term and late preterm infants are scattered over a broad range of facilities, many of which care for relatively few infants each year with NE. NER hospitals may be the best representation of those caring for encephalopathic infants in the “real world” and represent a generalizable view of HT as it occurs outside the academic sector or in a research setting. VON NER centers are heterogeneous in terms of size and numbers of infant records submitted. However, the participating nurseries were largely non-profit tertiary referral centers. Slightly more than half were teaching hospitals.

The UK TOBY Cooling Register also captures data on neonatal HT [26,27]. The TOBY Register started after the TOBY trial of HT closed enrollment, upon recognition that many physicians were offering HT out of the context of any trial [6,28]. It is a phase 4 study of the specific methods of the trial, with a narrower set of inclusion criteria than the VON NER. Comparison of information in the VON and the TOBY registries will be useful in understanding dissemination of HT when implemented strictly in accordance with a previous trial (TOBY) versus in a more broad clinical setting (VON NER).

### Limitations

Registries have important limitations with respect to RCTs. RCTs have strong internal validity, but often are focused on a relatively homogeneous group of patients from whom significant numbers are excluded at the cost of external validity or widespread generalizability. Registries aim for greater generalizability with populations relevant to all clinical settings. The nature of registry data limits clinicians from applying registry data to clinical decision-making. However, careful data collection and analyses of the NER, with oversight by the Steering Committee, aim to limit the potential for bias and misinterpretation of data. Awareness and recognition of bias in registry data adds to its heuristic value for planning clinical research or guiding NICU policies.

## Conclusion

A registry is well suited to the study of the heterogeneous population of NE infants and to the characterization of how interventions such as HT are implemented in clinical practice. This manuscript describes the methods and initial demographic results of the VON NER. Future manuscripts are planned on antecedents of NE, evaluation and treatment of neonatal seizure, optimal neuroimaging of NE infants, and hypothermia for HIE in routine practice.

## Competing interests

The VON NER is funded solely by the Vermont Oxford Network. The Vermont Oxford Network will pay the article processing charge. Drs. Horbar and Soll are employees of Vermont Oxford Network. The Vermont Oxford Network nor any of the authors will receive reimbursements, fees, funding, or salary as a result of the publication of this manuscript. On December 20, 2006 the Food and Drug Administration (FDA) approved the Olympic Cool-Cap® device to provide selective head cooling in infants with clinical evidence of moderate to severe hypoxic ischemic encephalopathy. The FDA has required that Olympic Medical Corporation provide Cool-Cap® users the opportunity to enroll cooled infants in a registry. Olympic Medical Corporation contracted with the Vermont Oxford Network to make its Registry available to Cool-Cap® users who are members of the Network. The Vermont Oxford Network provides Olympic Medical Corporation with special de-identified reports for these infants which Olympic Medical Corporation may choose to submit to the FDA for post marketing surveillance.

## Authors’ contributions

RP performed the primary analysis and interpretation of data and was responsible for primary manuscript preparation. PB participated in the study design and construction and reviewed the manuscript. EE performed statistical analysis and manuscript review. JH conceived of the study and participated in its design and coordination. MK performed the statistical analysis and reviewed the manuscript. TI participated in the study design and construction and reviewed the manuscript. KN participated in the study design and construction and reviewed the manuscript. TR participated in the study design and construction and reviewed the manuscript. RS participated in the study design and construction and reviewed the manuscript. All authors read and approved the final manuscript.

## Pre-publication history

The pre-publication history for this paper can be accessed here:

http://www.biomedcentral.com/1471-2431/12/84/prepub
